# Storage of Factor VIII Variants with Impaired von Willebrand Factor Binding in Weibel-Palade Bodies in Endothelial Cells

**DOI:** 10.1371/journal.pone.0024163

**Published:** 2011-08-31

**Authors:** Maartje van den Biggelaar, Eveline A. M. Bouwens, Jan Voorberg, Koen Mertens

**Affiliations:** 1 Department of Plasma Proteins, Sanquin Research, Amsterdam, The Netherlands; 2 Department of Pharmaceutics, Utrecht Institute for Pharmaceutical Sciences, Utrecht University, Utrecht, The Netherlands; 3 Landsteiner Laboratory of AMC and Sanquin, University of Amsterdam, Amsterdam, The Netherlands; University of Sao Paulo–USP, Brazil

## Abstract

**Background:**

Point mutations resulting in reduced factor VIII (FVIII) binding to von Willebrand factor (VWF) are an important cause of mild/moderate hemophilia A. Treatment includes desmopressin infusion, which concomitantly increases VWF and FVIII plasma levels, apparently from storage pools containing both proteins. The source of these VWF/FVIII co-storage pools and the mechanism of granule biogenesis are not fully understood.

**Methodology/Principal Findings:**

We studied intracellular trafficking of FVIII variants implicated in mild/moderate hemophilia A together with VWF in HEK293 cells and primary endothelial cells. The role of VWF binding was addressed using FVIII variants displaying reduced VWF interaction. Binding studies using purified FVIII proteins revealed moderate (Arg2150His, Del2201, Pro2300Ser) to severe (Tyr1680Phe, Ser2119Tyr) VWF binding defects. Expression studies in HEK293 cells and primary endothelial cells revealed that all FVIII variants were present within VWF-containing organelles. Quantitative studies showed that the relative amount of FVIII storage was independent of various mutations. Substantial amounts of FVIII variants are co-stored in VWF-containing storage organelles, presumably by virtue of their ability to interact with VWF at low pH.

**Conclusions:**

Our data suggest that the potential of FVIII co-storage with VWF is not affected in mild/moderate hemophilia A caused by reduced FVIII/VWF interaction in the circulation. These data support the hypothesis that Weibel-Palade bodies comprise the desmopressin-releasable FVIII storage pool *in vivo*.

## Introduction

Factor VIII (FVIII) is an essential cofactor in the factor Xa (FXa) generating complex by accelerating the factor IXa (FIXa) mediated-conversion of factor X (FX) into activated factor X (FXa) [1]. The FVIII translation product consists of a 19 amino acid signal peptide followed by a 2332 amino acid precursor protein that is organized in a distinct domain structure: A1-*a1*-A2-*a2*-B-*a3*-A3-C1-C2. Due to intracellular proteolytic processing, FVIII circulates in plasma as a heterodimer consisting of a 90–220 kDa heavy chain (A1-*a1*-A2-*a2*-B) and a 80 kDa light chain (*a3*-A3-C1-C2). The heavy chain and light chain of FVIII remain associated through a variety of interactions, some of which are metal-ion dependent [2]. In the circulation, FVIII travels in complex with its carrier protein von Willebrand factor (VWF), preventing premature clearance and proteolytic degradation of FVIII [1].

A defect in the gene encoding for FVIII results in the X-chromosome linked bleeding disorder hemophilia A. Large deletions, frame-shifts, premature stop codons or intron inversions are most commonly associated with severe hemophilia A and result in functional FVIII levels below 1% [3]. Severe hemophilia A patients are treated with on-demand or prophylactic protein replacement therapy using plasma derived or recombinant FVIII concentrates. Point mutations and small in-frame insertions or deletions in the FVIII gene generally result in a moderate or mild hemophilia A phenotype with circulating functional FVIII plasma levels between 1–5% and 5–30% respectively [3]. The molecular mechanisms that underlie moderate and mild hemophilia A include defects with respect to biosynthesis, impaired secretion, altered interaction with factor IXa, reduced binding to phospholipid membranes, impaired thrombin activation, impaired stability in the circulation or a reduced ability to associate with VWF in plasma [3], [4]. In addition to protein replacement therapy, mild or moderate hemophilia A patients can be treated with infusions of the vasopressin analogue desmopressin (DDAVP) [5], [6].

Administration of DDAVP releases both VWF and FVIII in the circulation [7]. The source of DDAVP-releasable VWF and FVIII has not been established. However, several lines of evidence suggest that FVIII and VWF are synthesized and stored within the same cell [8], [9]. This view is supported by the observation that *in vitro* co-expression of VWF and FVIII results in storage of FVIII in VWF-containing organelles [10]-[15]. Lung microvascular endothelial cells and liver sinusoidal endothelial cells both synthesize VWF and FVIII *in vivo* [16]–[18]. Recently, it has been shown that endothelial cells from several vascular beds, including the hepatic sinusoid and pulmonary vascular circulation, can synthesize and secrete FVIII [19].

We have previously demonstrated that FVIII trafficking to VWF-containing storage organelles is independent of high-affinity binding to VWF [13], [15]. VWF 2N variants that do not bind FVIII are still able to induce FVIII storage in WPBs [15], providing a rationale for the observed DDAVP-induced release of FVIII and VWF in type 2N VWD patients [20]. It remains unknown whether, in addition to type 2N VWD patients, co-storage of FVIII and VWF may also underlie the DDAVP-mediated increase of FVIII plasma levels in patients suffering from mild/moderate hemophilia. The aim of this study was therefore to analyze VWF co-storage for a panel of FVIII variants associated with mild/moderate hemophilia A due to reduced binding to VWF. For these studies, we selected 5 FVIII variants that have been established to cause mild to moderate hemophilia A due to reduced binding to VWF [21]–[24]. We analyzed targeting of these FVIII variants to VWF-containing granules in heterologous HEK293 cells as well as in primary endothelial cells. Our results demonstrate that, despite impaired complex assembly with VWF, all FVIII variants retain their capability to traffic to VWF-containing organelles. These data support the hypothesis that FVIII-containing WPBs represent a desmopressin-releasable storage pool of VWF and FVIII *in vivo*.

## Results

### Expression of FVIII-YFP variants in HEK293 cells

To address the role of amino acid substitutions that cause mild/moderate hemophilia A in the intracellular trafficking of FVIII, we have created 5 FVIII variants in B-domain deleted FVIII-YFP that have been established to cause mild to moderate hemophilia A due to reduced binding to VWF [21]–[24], including single Tyr1680Phe, Ser2119Tyr, Arg2150His, Del2201 or Pro2300Ser substitutions. The effect of the hemophilic replacements on secretion of FVIII was studied. FVIII-YFP variants were expressed in HEK293 cells stably expressing VWF-CFP and FVIII levels in conditioned medium were quantified based on heterodimer ELISA and chromogenic activity (Table 1). Transient expression of wild type FVIII-YFP resulted in significant levels of FVIII in conditioned medium (5.0±0.4 pmol FVIII:Ag/1×10^6^ cells per 72 hours) (Table 1). Levels of FVIII-YFP variants carrying a Tyr1680Phe and Ser2119Tyr replacement or a deletion of Ala2201 were approximately 50% reduced whereas levels of FVIII-YFP variants carrying an Arg2150His or Pro2300Ser were more than 10-fold reduced (Table 1). None of the variants showed an apparent secretion defect (Figure 3, see below). The levels of VWF were not affected (6.7±0.8 pmol VWF/1×10^6^ cells per 72 hours).

**Table 1:** Specific activity and production levels of FVIII variants.

FVIII-YFP	Production levels		Specific activity of
variant			purified variants
	pmol FVIII/1×10^6^ cells/72 hours		FVIII activity (U)/
	antigen	activity	mg protein
wild type	5.0±0.3	4.1±0.4	3.2±0.3×10^3^
Tyr1680Phe	3.9±0.4	2.3±0.5	2.1±0.2×10^3^
Ser2119Tyr	3.2±0.4	2.0±0.2	3.6±0.3×10^3^
Arg2150His	0.3±0.1	0.3±0.1	1.2±0.1×10^3^
Del2201	2.7±0.1	2.0±0.1	2.1±0.1×10^3^
Pro2300Ser	0.3±0.1	0.3±0.1	3.4±0.2×10^3^

Production levels of FVIII: Ag were quantified by heterodimer ELISA using CLB-CAg9 and CLB-CAg117 and FVIII activity levels were quantified by chromogenic activity. Specific activity of purified FVIII-YFP variants was evaluated as the FVIII activity as determined by chromogenic assay (in U) per mg of protein as determined by Bradford assay. Each value represents the mean ± SD of at least three measurements.

### Purification of FVIII-YFP variants produced by HEK293 cells

**Figure 1:**
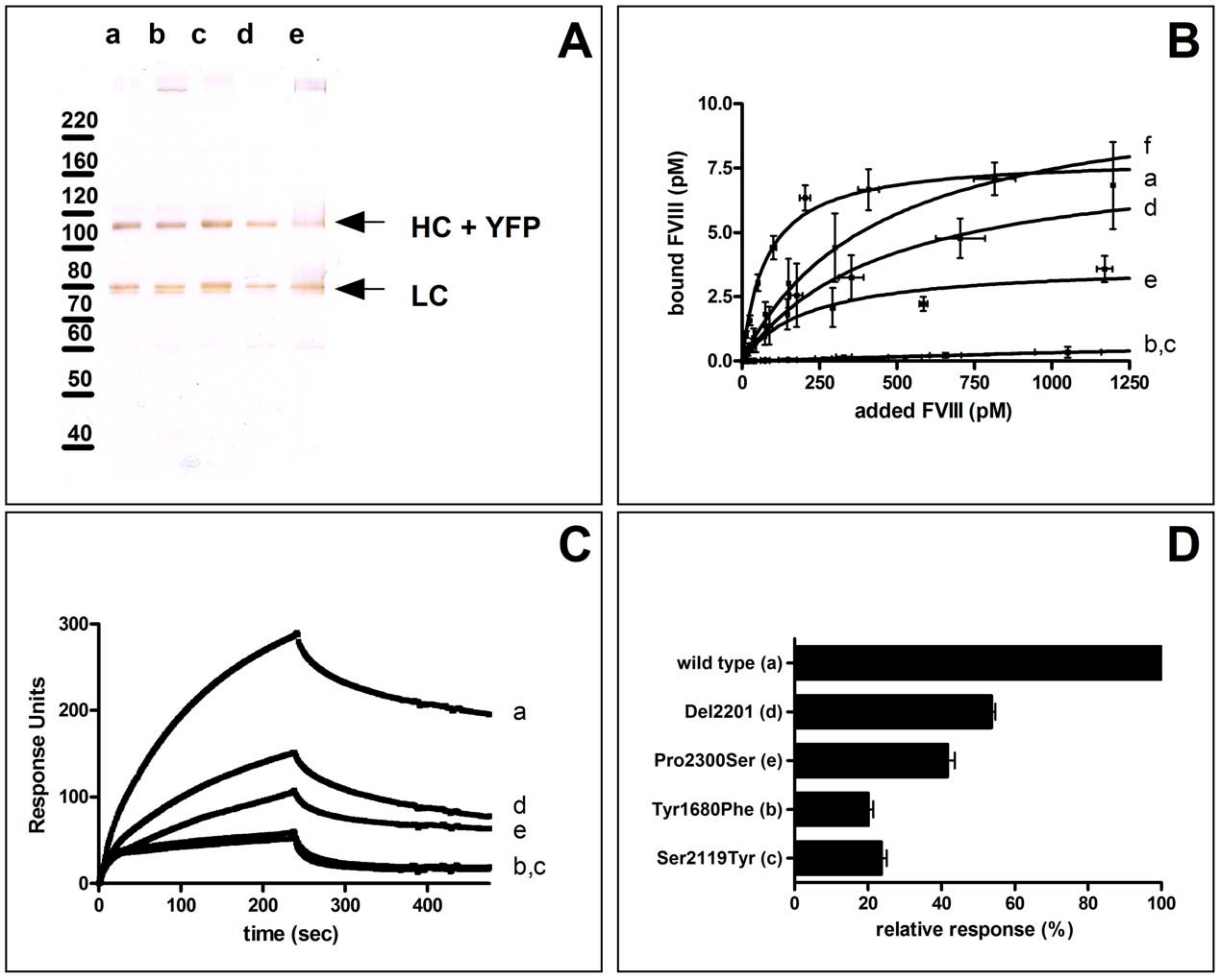
Binding of purified fluorescent FVIII-YFP variants to VWF. Letters represent wild type FVIII-YFP (a) Tyr1680Phe, (b) Ser2119Tyr, (c) Del2201 (d), Pro2300Ser (e) and Arg2150His (f). (A) Purified protein preparations were analyzed by 7.5% sodium dodecylsulfate polyacrylamide gel electrophoresis under reducing conditions followed by silver staining. HC  =  heavy chain. LC =  light chain. YFP  =  yellow fluorescent protein. (B) Pseudo-equilibrium binding of FVIII-YFP variants to VWF using an ELISA-based format was performed. The amount of FVIII bound to VWF was plotted against the amount of FVIII added. Data were analyzed by non-linear regression using a standard hyperbola. Each value represents the mean of three experiments. (C) SPR analysis of FVIII binding to VWF. FVIII variants were passed over a chip to which 22 fmol/mm^2^ recombinant wild type VWF was coupled in a buffer containing 150 mM NaCl, 5 mM CaCl_2_, 5% (v/v) Glycerol, 0.005% (v/v) Tween 20 and 20 mM Hepes (pH 7.4) at 25°C with a flow rate of 20 µL/min. Association and dissociation phase were both followed for 240 seconds. Shown is the average curve of three injections of 2.5 nM of (each) FVIII variant. (D) Percentage of binding was calculated relative to wild type FVIII after 235 seconds of association. Values represent the mean ± SEM of at least 6 injections (1–10 nM FVIII variants).

To address the ability of the FVIII variants to associate with VWF, FVIII-YFP variants were purified from the conditioned medium of HEK293 cell-lines expressing FVIII-YFP variants (in absence of VWF) using immunoaffinity chromatography. Due to low production levels, the Arg2150His variant could only be purified partially. After purification, the values for the specific activity of all other variants were at least >2000 U/mg (Table 1). SDS-PAGE analysis demonstrated that all these FVIII variants were processed and secreted predominantly as a heterodimer with virtual absence of single chain FVIII (Figure 1A).

### Pseudo-equilibrium FVIII/VWF binding

Binding of FVIII-YFP variants, including the partially purified Arg2150His variant, to VWF was analyzed under pseudo-equilibrium conditions (Figure 1B) [13]. As described previously [13], FVIII-YFP readily bound to VWF with high affinity in the subnanomolar range (Figure 1B). All FVIII-YFP variants demonstrated a binding defect, in the following qualitative ranking: Arg2150His>Del2201>Pro2300Ser>Tyr1680Phe = Ser2119Tyr.While variants carrying a Tyr1680Phe or Ser2119Tyr replacement demonstrated a severe reduction in VWF binding, appreciable binding was observed for the variants carrying an Arg2150His and Pro2300Ser replacement or deletion of Ala2201 (Figure 1B).

### Surface plasmon resonance analysis of FVIII/VWF binding

We further used Surface Plasmon resonance (SPR) analysis to study binding of individual FVIII-YFP to purified recombinant VWF containing high molecular weight multimers. The Arg2150His variant could not be analyzed as this variant was only partially purified. Representative SPR experiments are shown in Figure 1C and data are summarized in Figure 1D. SPR data demonstrated that wild type FVIII-YFP readily bound to VWF (Figure 1C curve a). All FVIII-YFP variants showed reduced binding to VWF (Figure 1C curve b–e). Variants carrying a Tyr1680Phe and Ser2119Tyr replacement demonstrated a severe reduction in VWF binding. For these variants, some residual binding occurred which was rapidly lost during the dissociation phase (Figure 1C curve b,c). Variants carrying a Pro2300Ser replacement or a deletion of Ala2201 demonstrated a binding defect that was less pronounced (Figure 1C curve d,e). In addition, binding proved partially irreversible, presumably due to rapid rebinding of FVIII to the immobilized VWF immediately following dissociation. Rapid rebinding often implies that association is mass transport limited, which prohibits calculation of reliable binding kinetics [25]. Therefore, SPR data were used to qualitatively compare the different variants (Figure 1D). In agreement with the binding assay under pseudo-equilibrium conditions (Figure 1B), all FVIII-YFP variants demonstrated a binding defect, in the following qualitative ranking: Del2201>Pro2300Ser>Tyr1680Phe = Ser2119Tyr.

### FVIII-VWF co-trafficking in HEK293 cells

**Figure 2:**
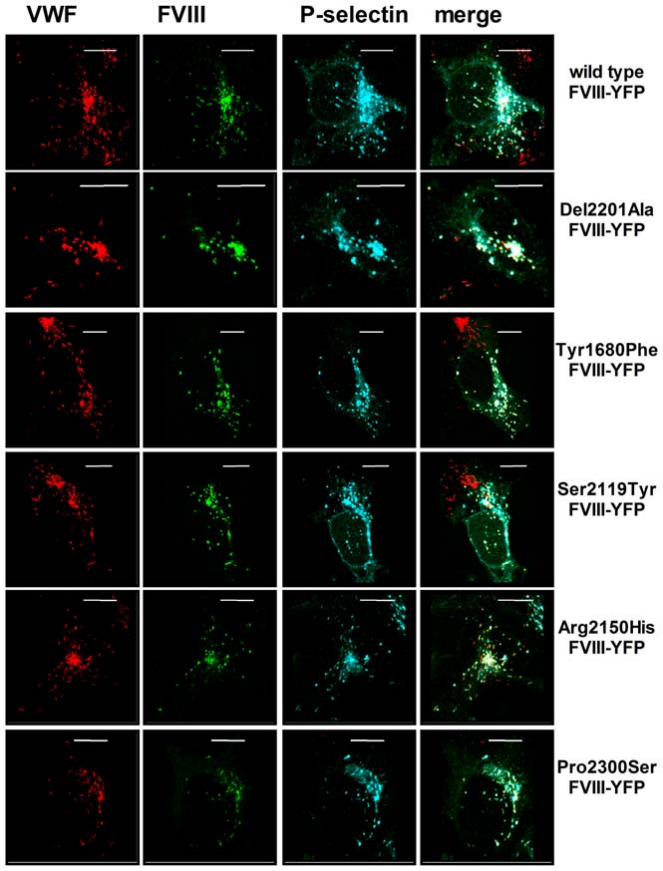
Intracellular localization of FVIII-YFP variants, P-selectin and fluorescent VWF-CFP. HEK293 cells stably expressing VWF-CFP were transfected with P-selectin and FVIII-YFP variants. Cells were stained for P-selectin using polyclonal anti-human CD62P antibody. VWF-CFP, FVIII-YFP and P-selectin are shown in red, green and blue respectively. Triple fluorescent detection is shown in the colour merges. Shown are representative 3-dimensional projections of Z-stacks (0.4 µm). The scale bar represents 10 µm.

We subsequently addressed the subcellular localization of FVIII variants in HEK293 cells. Wild type FVIII-YFP staining is associated with the secretory pathway at the level of the *trans*-Golgi network and endoplasmatic reticulum [13]. Expression of hemophilic FVIII-YFP variants resulted in a similar localization (data not shown). As reported before [13], FVIII-YFP was observed in VWF-containing organelles in cells expressing VWF-CFP (Figure 2). Co-expression studies with P-selectin revealed that the VWF/FVIII-containing organelles retained the ability to recruit P-selectin. FVIII-YFP variants that demonstrate a moderate (Del2201) or severely reduced binding to VWF (Tyr1680Phe and Ser2119Tyr) were still able to co-traffic to VWF/P-selectin-containing granules (Figure 2). In addition, the FVIII-YFP variants carrying an Arg2150His or Pro2300Ser are stored in VWF/P-selectin-containing granules despite a moderate reduction in VWF binding and a more than 10-fold reduction in FVIII levels in conditioned medium (Figure 2).

### Storage of FVIII-YFP variants in VWF-containing granules in HEK293 cells

**Figure 3:**
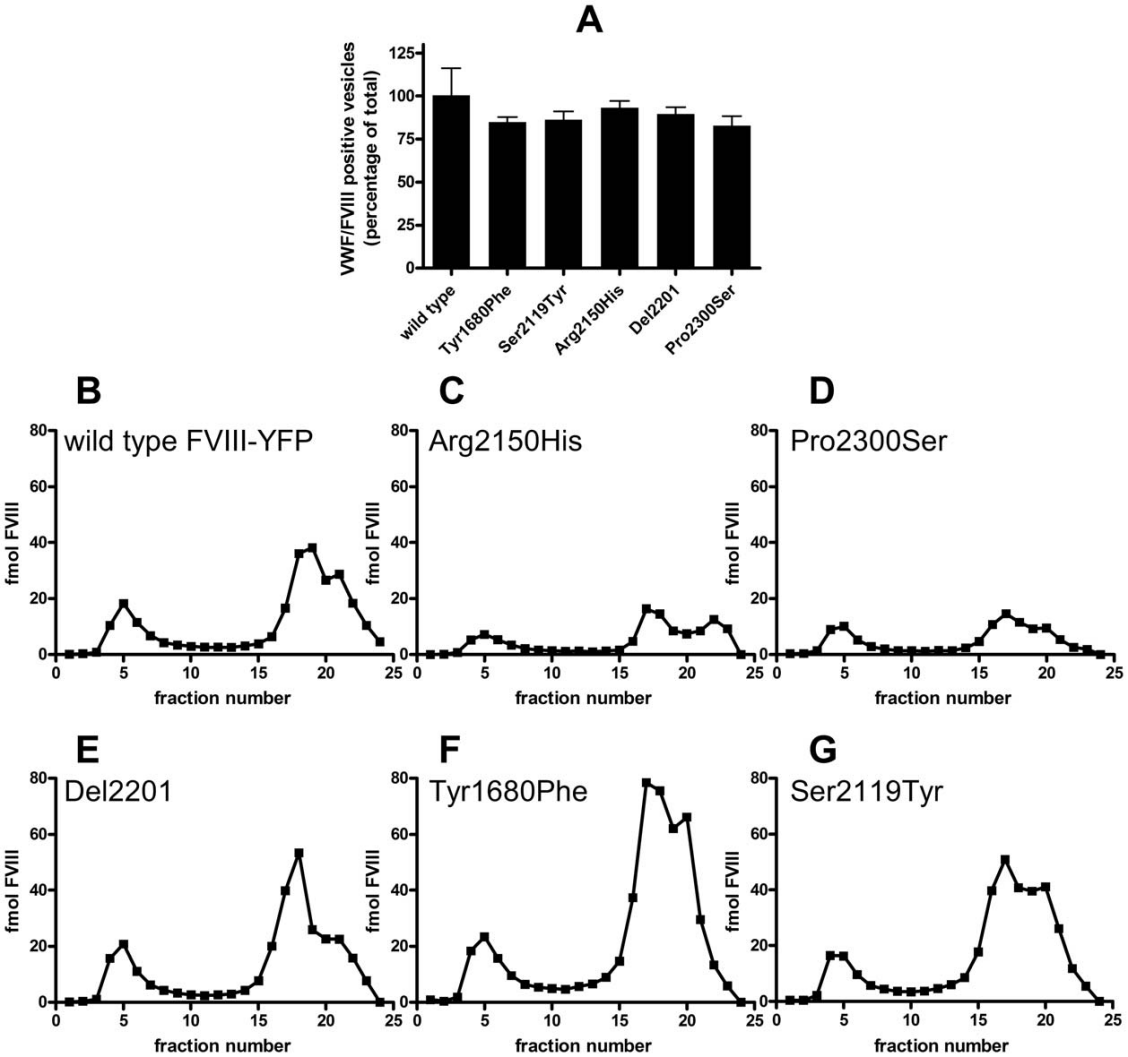
Quantitative analysis of storage of FVIII variants in VWF-CFP-containing organelles. HEK293 cells stably expressing VWF-CFP were transfected with FVIII-YFP variants. (A) Cells were analyzed by Confocal Laser Scanning Microscopy. Z-stacks of 0.4 µm sections of individual cells were acquired. A 3-dimensional reconstruction was created and the number of WPBs containing VWF and VWF/FVIII was quantified. Shown is the mean and standard deviation of at least 7 individual cells per FVIII variant. (B–G) Representative subcellular fractionations are shown. FVIII antigen was quantified in individual fractions by anti-FVIII light chain ELISA. The amount of FVIII light chain in fmol is plotted on the Y-axis against the fraction number on the X-axis.

So far, we have analyzed targeting of FVIII and VWF on a qualitative level. Our findings indicate that FVIII variants displaying a strongly reduced affinity for VWF can enter VWF-containing granules. We subsequently explored whether the amount of FVIII stored within VWF-containing granules depends on the affinity of FVIII for VWF. To address FVIII storage in VWF-containing granules in a more quantitative manner, we analyzed the amount of VWF-CFP containing vesicles that also contained FVIII. We found that independent of the amino acid replacement, 80–100% of VWF-containing vesicles contained FVIII-YFP (Figure 3A). This observation indicates that targeting of FVIII variants to VWF-containing vesicles is independent of their affinity for VWF. In addition, we performed subcellular fractionations followed by density gradient centrifugation. Homogenates of transfected HEK293 cells were subjected to density gradient centrifugation and the amount of VWF and FVIII in the various fractions was determined by ELISA. Representative fractionations are shown in Figure 3B-G. The dense fraction containing the WPB-like organelles corresponds with fractions 4–9, whereas the second peak contains the subcellular fractions derived of organelles of the secretory pathway (ER, Golgi, *trans-*Golgi network) and constitutively released vesicles [26]. For wild type FVIII-YFP, 21±6% of FVIII-YFP antigen and 16±5% of VWF-CFP antigen was found in the dense fraction of the cell (Table 2). To exclude that presence of the fluorescent YFP and CFP moieties influences targeting, subcellular fractionations were also performed using untagged FVIII and VWF variants. For untagged FVIIIdB, 17% was targeted to the dense fraction of the cell containing wild type VWF (data not shown). This indicates that the YFP/CFP moieties do not contribute to targeting of FVIII and VWF. Storage of all FVIII-YFP variants in VWF-CFP containing granules was quantified. As expected, total intracellular FVIII antigen levels were reduced for the variants carrying an Arg2150His or Pro2300Ser replacement (Figure 3C,D). However, despite the reduced FVIII levels, a significant portion of the intracellular FVIII antigen was stored within the dense fraction of the cell. In addition, FVIII-YFP variants that display a moderate (Del2201) or severe reduction in VWF binding (Tyr1680Phe or Ser2119Tyr), are clearly stored in the fraction corresponding with VWF-containing granules (Figure 3E,F,G). These data demonstrate that substantial amounts of mild/moderate hemophilia A causing FVIII variants can be stored in VWF-containing storage organelles (Table 2).

**Table 2:** Subcellular fractionation of transfected HEK293 cells.

FVIII-YFP	Percentage VWF	Percentage FVIII
variant		
	fractions 4–9	fractions 4–9
wild type	16±5	21±6
Tyr1680Phe	16±5	14±6
Ser2119Tyr	19±6	16±4
Arg2150His	18±4	16±8
Del2201	16±5	17±4
Pro2300Ser	21±4	18±11

HEK293 cells stably expressing VWF-CFP were transfected with FVIII-YFP variants. FVIII antigen was quantified by anti-FVIII light chain ELISA. VWF antigen was quantified by ELISA. The amount of FVIII and VWF antigen in the pooled fractions 4–9 is divided by the total amount of FVIII and VWF antigen in the pooled fractions 1–25. The values represent the mean ± SD of three measurements.

### FVIII-VWF co-trafficking in Blood Outgrowth Endothelial Cells (BOECs)

**Figure 4:**
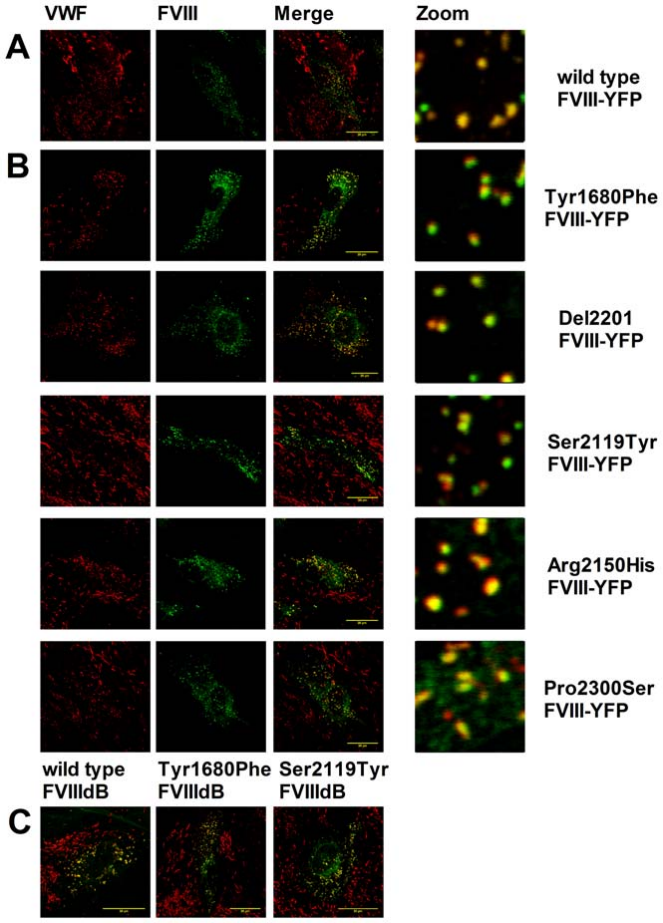
Co-trafficking of FVIII variants to Weibel-Palade bodies in endothelial cells. Human BOEC passage 8 were transduced with lentiviral vectors encoding (A) FVIII-YFP, (B) FVIII-YFP variants carrying Tyr1680Phe, Del 2201, Ser2119Tyr, Arg2150His and Pro2300Ser replacements or (C) FVIIIdB wild type and variants carrying Tyr1680Phe or Ser2119Tyr replacements. Cells were stained for VWF using monoclonal antibody CLB-RAg20, shown in red (A,B,C) and for FVIIIdB (variants) (C) using FITC-labelled monoclonal antibody EL14 IgG4. FVIII-YFP and FVIII dB (variants) are shown in green. Double fluorescent detection is shown in the colour merges. Shown are representative 3-dimensional projections of Z-stacks (0.4 µm). The scale bar represents 20 µm.

To validate the use of HEK293 cells as a model system, we also analyzed trafficking of FVIII-YFP variants in genuine endothelial cells. Previous studies have shown that BOEC provide an excellent model for studying the biosynthesis of VWF as well as targeting of FVIII to WPBs [14], [27]. As was demonstrated for HEK293 cells, all FVIII-YFP variants were stored in organelles that also contained VWF. We conclude that FVIII trafficking to WPBs in BOECs is independent of the absence of high-affinity interaction with VWF (Figure 4A,B). To exclude the possibility that the YFP moiety contributes to FVIII targeting to WPBs, we also studied trafficking of Tyr1680Phe and Ser2119Tyr variants in a FVIIIdB background. Again, all FVIII variants were co-stored with VWF in WPBs (Figure 4C). Upon FVIII co-trafficking, WPBs lose their elongated shape and become spherical [14], [15]. We have previously shown that this shape change is specific for the presence of FVIII as co-transfection of other WPB residents, including VWF-YFP and P-selectin, do not result in a shape change of the WPB [15]. Interestingly, the transition from elongated to spherical WPBs was observed for all FVIII variants, independent of their affinity for VWF and presence of the YFP moiety (Figure 4).

### FVIII-YFP Tyr1680Phe is co-stored with the VWF type 2N variant VWF-CFP Arg763Gly

**Figure 5:**
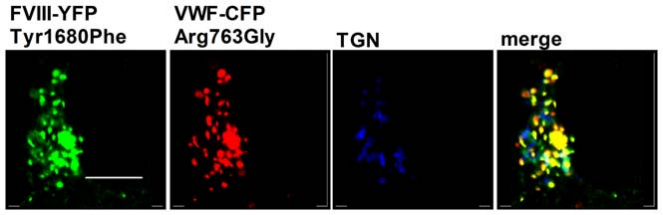
Co-storage of FVIII-YFP Tyr1680Phe with VWF type 2N variant VWF-CFP Arg763Gly. HEK293 cells stably expressing FVIII-YFP Tyr1680Phe were transfected with VWF-CFP Arg763Gly. Cells were stained for the *trans*-Golgi network using polyclonal antibody TGN46. Triple fluorescent detection is shown in the colour merge. Shown is a representative 3-dimensional projection of Z-stack (0.4 µm). The scale bar represents 10 µm.

So far, our results show that targeting of FVIII variants to WPBs is not affected by a reduced binding of these variants to VWF. However, for most variants some residual binding to VWF is observed. To more precisely define whether FVIII binding to VWF is required for its targeting to WPBs, we used the previously described VWF type 2N variant (VWF-CFP Arg763Gly) [15] in conjunction with FVIII-YFP Tyr1680Phe. Despite a negligible interaction in our SPR experiments (data not shown), FVIII-YFP Tyr1680Phe was stored together with VWF-CFP Arg763Gly in HEK293 cells (Figure 5).

### Binding of FVIII-YFP to VWF is pH dependent

**Figure 6:**
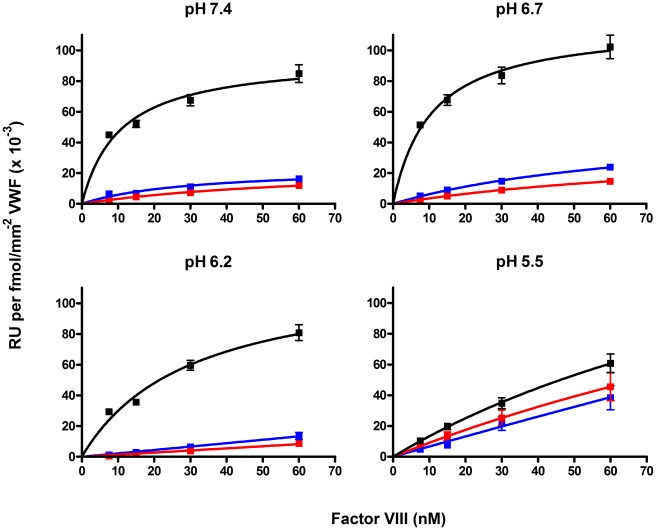
pH dependent binding of FVIII-YFP variants to VWF. FVIII-YFP (black), FVIII-YFP Tyr1680Phe (red) and FVIII-YFP Ser2119Tyr (blue) (7.5–90 nM) were passed over various densities (23–39 fmol/mm^2^) of immobilized recombinant wild type VWF in buffers containing 150 mM NaCl, 5 mM CaCl_2_, 0.005% (v/v) Tween 20 and 20 mM Hepes (pH 7.4 and 6.7) or 20 mM MES (pH 6.2 and 5.5). The response at 235 seconds of association was expressed per amount of VWF immobilized on the SPR chip and plotted against the concentration of FVIII-YFP variants. Values represent the mean ± standard deviation for the three different VWF densities.

In the previous paragraphs we show that the impaired binding of FVIII variants to VWF does not abolish trafficking to WPBs. Sorting of proteins to storage organelles occurs at the levels of the *trans*-Golgi network (TGN). Acidification of vesicles along the secretory pathway provides the driving force of sorting of secretory hormones [28]. During formation of storage granules the intravesicular pH decreases from 6.2 to 5.5 [28]. This prompted us to analyze the effect of pH on the interaction between VWF and FVIII. SPR binding experiments to VWF were performed for wild type FVIII-YFP, the Tyr1680Phe and Ser2119Tyr variant in a pH ranging from 7.4 to 5.5. Binding of wild type FVIII-YFP was similar over the pH range 7.4 to 6.2, but decreased at pH 5.5 (Figure 6). In agreement with the data presented in Figure 1, binding of the FVIII-YFP variants Tyr1680Phe and Ser2119Tyr to VWF was severely reduced when compared to wild type FVIII-YFP at the pH range from 7.4 to 6.2 (Figure 6). Unexpectedly, binding of the Tyr1680Phe and Ser2119Tyr variants was increased at pH 5.5, and proved only slightly lower than that of wild type FVIII-YFP (Figure 6).

## Discussion

Most mild and moderate hemophilia A patients can be effectively treated with DDAVP [5]. Treatment with DDAVP results in a concomitant increase of VWF and FVIII in the circulation, presumably by the release of storage pools that contain both FVIII and VWF. The formation of DDAVP-releasable co-storage pools requires endogenous synthesis of both FVIII and VWF. This is illustrated by the inability of DDAVP to mediate a rise in circulating FVIII levels in patients with severe (type 3) von Willebrand's disease or severe hemophilia A [8], [9]. Restoring FVIII or VWF plasma levels to therapeutic levels by prophylactic protein replacement therapy, does not result in the formation of a DDAVP releasable co-storage pool in these patients [8], [9]. Apparently, DDAVP-releasable storage pools can not be restored by uptake of FVIII and VWF from the circulation.

While it is generally recognized that DDAVP releases VWF from WPBs, the origin and nature of the DDAVP-sensitive storage compartment of FVIII has not yet been defined. We and others have proposed that the DDAVP-induced rise of both FVIII and VWF argues for co-storage of both these proteins in WPBs. Pertinent to this point is our recent observation that VWF type 2N variants, despite a markedly decreased ability to bind to FVIII, drive co-trafficking of FVIII to VWF-containing granules [15]. In addition, we have previously demonstrated that the Tyr1680Phe FVIII variant is co-stored with VWF in WPBs despite its severely reduced interaction with VWF [13]. This raises the question as to whether VWF co-storage of FVIII variants displaying a reduced ability to associate with VWF represents a general phenomenon in mild/moderate hemophilia A. We have therefore extended our initial observation regarding the Tyr1680Phe variant to a larger panel of mild/moderate hemophilia A causing FVIII variants, including amino acid replacements in the FVIII C1 and C2 domains. In addition, we now have used a quantitative approach to assess trafficking of FVIII to VWF-containing granules in HEK293 cells. Moreover, we addressed co-trafficking of GFP-tagged as well as untagged FVIII variants in endothelial cells, with particular reference to the morphology of FVIII-containing WPBs.

We demonstrate that point mutations in the C1 and C2 domains of FVIII can have diverse effects on its synthesis, secretion and ability to bind to VWF without loss in cofactor function, in agreement with previously published data [21]. The ranking of VWF binding is the following: wild type>Arg2150His>Del2201>Pro2300Ser>Ser2119Tyr = Tyr1680Phe (Figure 1). Remarkably, notwithstanding their reduced capacity to bind to VWF and/or reduced levels of synthesis, substantial amounts of moderate/mild hemophilia A causing FVIII variants can be stored in VWF-containing granules (Figures 2, 3, 4). Our quantitative studies showed that, independent of the amino acid replacement, relative amounts of FVIII stored in VWF-containing granules are similar (Figure 3). Assuming that co-expression and co-storage of FVIII and VWF does occur *in vivo*, our data provide a molecular explanation for the fact that hemophilia A patients suffering from impaired complex assembly of FVIII and VWF in the circulation can be effectively treated with DDAVP. In particular, hemophilia A patients carrying a Tyr1680Phe, Ser2119Tyr, Arg2150His replacement or deletion of Ala2201, respond to DDAVP treatment by a concomitant increase of FVIII and VWF [22], [29]. In previous studies FVIII has been expressed in VWF-containing α-granules in mice. In this model, the benefit of FVIII release concomitant with platelet activation was convincingly demonstrated [30], [31]. Recently, targeting of FVIII to WPBs has been shown to restore hemostasis in a mouse model of hemophilia A [32]. This finding further emphasizes the hemostatic potential of DDAVP-induced FVIII release from WPBs.

The mechanism of FVIII/VWF co-storage in WPBs has not been elucidated so far. It seems logical to suppose that FVIII targets to the WPBs by virtue of a direct interaction with VWF within the secretory pathway. In cells that contain a regulated secretory pathway, the pH decreases from pH 7.4 in the ER, to pH 6.2 in the Golgi, to pH 5.5 in mature secretory granules [28]. This acidification process is essential for sorting and processing of regulated secretory hormones [28]. In endothelial cells, a decreasing pH along the secretory pathway has been postulated to mediate WPB biogenesis by coordinating the disulfide-linked assembly of VWF multimers and their tubular packing [33]. At pH 6.2, intersubunit disulfide bond formation between VWF propeptide and D'D3 dimers results in the generation of helical tubules [33] which most likely underlies the biogenesis of elongated WPBs [34]. It has been suggested that histidine residues in the propeptide function as pH sensors and control the disulfide bond formation within D'D3 [35].

We observed that the FVIII Tyr1680Phe and Ser2119Tyr variants bind better to VWF under acidic conditions (Figure 6). This indicates that FVIII can bind to VWF in multiple ways, including high-affinity interaction with a pH optimum of 6.7 and low-affinity interaction with a pH optimum of 5.5 or lower. At first sight, these data suggest that pH-dependent binding of FVIII and VWF along the secretory pathway may drive co-targeting of FVIII to WPBs suggesting that ‘histidine switch’ may also control entry of FVIII in WPBs. However, targeting of proteins to the secretory pathway is thought to occur at the *trans-*Golgi network where intracellular conditions are intermediate between ER and secretory organelles, pH 6.2. In endothelial cells, the pH has been described to be 5.45±0.26 for resting WPBs and 6.16±0.45 for newly formed WPBs [36]. The latter WPBs, which presumably have just been pinched off from the *trans-*Golgi network, thus are just in the pH range wherein the changes in pH-dependent VWF-FVIII interaction occur. While this would explain that FVIII and VWF may be associated within WPBs, it remains unclear whether or not this would be sufficient for association of FVIII and VWF within the secretory pathway during WPB biogenesis. Our data seem equally compatible with the existence of an alternative, VWF-independent targeting mechanism. It remains difficult to explain why the combination of the FVIII Tyr1680Phe variant with a VWF type 2N variant that is severely defective in interacting with FVIII [15], still leads to co-storage with VWF (Figure 5). Both the pH-dependent FVIII-VWF association and the possibility of a VWF-independent targeting mechanism remain intriguing issues for further study.

## Materials and Methods

### Plasmid mutagenesis

Construction of wild type VWF [13], VWF-CFP with the Cyan Fluorescent Protein moiety replacing the A2 domain and VWF-CFP Arg763Gly [13], B-domain deleted (del 746–1639) FVIII-YFP with the Yellow Fluorescent Protein moiety replacing the B-domain (FVIII-YFP) [13] and P-selectin [37] in pcDNA3.1(+) have been described previously. Point mutations and deletions in FVIII-YFP were introduced by Quick Change mutagenesis™ (Table S1). B-domain deleted FVIII variants lacking the YFP moiety (FVIIIdB) were created by removal of the YFP moiety by Quick Change mutagenesis™ (Table S1). FVIII-YFP variants and B-domain deleted FVIII variants were ligated into the lentiviral self-inactivating vector under control of the CAG promoter consisting of the chicken β-actin promoter, CMV enhancers and a large synthetic intron [14] using NheI and NotI. The coding regions of all constructs were verified by sequence analysis. Sequence reactions were performed with BigDye Terminator Sequencing kit (Applied Biosystems, Foster City, CA, USA).

### Expression and purification of recombinant proteins

HEK293 cells (CRL-1573) (ATCC, Manassas, VA, USA) were grown in DMEM-F12 medium supplemented with 10% FCS, 100 units/ml penicillin and 100 µg/ml streptomycin (BioWhittacker, Verviers, Belgium). HEK293 cell-lines, stably expressing recombinant protein were produced as described [38]. Recombinant VWF containing high molecular weight multimers and FVIII variants were purified and analyzed as described [13].

### Binding of FVIII-YFP variants to VWF

Pseudo-equilibrium binding of FVIII variants to VWF using an ELISA-based format was performed and analyzed as described [13]. Association and dissociation of FVIII variants to wild type VWF was assessed by Surface Plasmon Resonance (SPR) analysis employing a BIAcore 3000 biosensor (Biacore AB, Uppsala, Sweden) [15]. Recombinant wild type VWF, VWF-CFP or VWF-CFP Arg763Gly (22 fmol/mm^2^) was immobilized onto a CM5 sensor chip using the amine coupling method according to the manufacturer's instructions. Binding to coated channels was corrected for binding in absence of VWF. Varying concentrations (1–15 nM) of FVIII-YFP variants were passed over the immobilized VWF in a buffer containing 150 mM NaCl, 5 mM CaCl_2_, 5% (v/v) Glycerol, 0.005% (v/v) Tween 20 and 20 mM Hepes (pH 7.4) at 25°C with a flow rate of 20 µL/min. The sensor chip surface was regenerated using the same buffer containing 1 M NaCl. To study the influence of pH on the interaction between VWF and FVIII (variants), wild type VWF was immobilized onto a CM5 sensor chip at various densities (23–39 fmol/mm^2^) and FVIII-YFP variants (Tyr1680Phe and Ser2119Tyr) were passed over the immobilized wild type VWF in buffers containing 150 mM NaCl, 5 mM CaCl_2_, 0.005% (v/v) Tween 20 and 20 mM Hepes (pH 7.4 or 6.7) or 20 mM MES (pH 6.2 or 5.5).

### Expression levels of FVIII-YFP variants

HEK293 cells stably expressing VWF-CFP were transfected using the calcium phosphate co-precipitation method essentially as described [39]. Briefly, 2.5×10^5^ cells/10 cm^2^ were seeded 24 hours before transfection on a gelatine-coated culture surface. Medium was refreshed 4 hours before transfection. Cells were transfected using 5 µg of plasmid DNA. Medium was refreshed 16 hours after transfection and cells were grown for an additional 72 hours. Antigen levels were determined by heterodimer ELISA [40] using CLB-CAg9 and CLB-CAg117. Activity levels were determined by chromogenic assay. Human pooled plasma from at least 30 donors, calibrated against the 5^th^ International Standard for FVIII and VWF in plasma (WHO 02/150), was used as a standard.

### Immunofluorescence analysis

HEK293 cells stably expressing VWF-CFP or FVIII-YFP Tyr1680Phe were transfected using the calcium phosphate co-precipitation method [39]. Blood outgrowth endothelial cells (BOECs) were isolated as described previously [14] from 50 ml of venous blood that was drawn from healthy anonymous volunteers, with written permission, in accordance with Dutch regulations and approval from Sanquin Ethical Advisory Board. Lentiviral preparations of FVIII variants were produced and BOECs were transduced as described [14]. Cells were prepared for immunofluorescence as described before [41]. Rabbit polyclonal antibody anti-human CD62-P (BD PharMingen, San Diego, CA, USA) was used to visualize P-selectin and sheep polyclonal antibody TGN46 (Serotec, Oxford, United Kingdom) was used to stain the *trans*-Golgi network. VWF was stained using monoclonal antibody CLB-RAg20 and FVIIIdB (variants) were stained using FITC-labeled human monoclonal antibody EL14 IgG4 [15]. Alexa-594 and Alexa-633- conjugated secondary antibodies were from Invitrogen (Breda, the Netherlands). Confocal Laser Scanning Microscopy was performed using a Zeiss LSM510 (Carl Zeiss, Heidelberg, Germany). For immunohistochemical analysis results were analyzed using Zeiss LSM510 version 4.0 software (Carl Zeiss, Heidelberg, Germany) or Image J (freely available through http://rsbweb.nih.gov/ij/). To obtain 3-dimensional images, Z-stacks of 0.4 µm sections of individual cells were acquired. A 3-dimensional reconstruction was created with Image Pro Plus 6.0 (Media Cybernetics, Breda, the Netherlands) and used to calculate the number of Weibel-Palade bodies within individual cells. In order to separate narrowly connected Weibel-Palade bodies, the 3D Watershed filter (threshold 10%) was applied. The number of Weibel-Palade bodies containing VWF and VWF/FVIII was quantified by using the volume measurements software in the 3D constructor module. Results were analyzed using GraphPad Prism 4 software.

### Subcellular fractionation

HEK293 cells stably expressing VWF-CFP or wild type VWF were transfected using 87.5 µg plasmid DNA per 175 cm^2^ flask as described above. Per FVIII variant, two 175 cm^2^ culture flasks were transfected. Subcellular fractionation using Percoll density gradient centrifugation was performed as described with minor modifications [42]. Briefly, cells were homogenized by 20 strokes in a ball-bearing homogenizer (Isobiotec, Heidelberg, Germany) with a 14 micron clearance. The homogenate was loaded on a Percoll gradient and centrifuged for 30 minutes at 100,000 *g* and 4°C. Fractions (1.25 ml) were collected from the bottom and stored at −20 °C. FVIII antigen was quantified by anti-light chain ELISA as described above. VWF antigen was quantified by ELISA essentially as described before [43]. Individual fractions were measured as well as pooled fractions 4–9 (dense fractions) and pooled fractions 1–25 (total).

## Supporting Information

Table S1 QuickChange Mutagenesis primers.
(DOC)
