# Supplementary material for: Storage of Factor VIII Variants with Impaired von Willebrand Factor Binding in Weibel-Palade Bodies in Endothelial Cells
Source: PLoS One. 2011 Aug 31;6(8):e24163. doi: 10.1371/journal.pone.0024163 (PMC3166073; doi:10.1371/journal.pone.0024163)
Supplement: Table S1 — QuickChange Mutagenesis primers. (DOC) [file pone.0024163.s001.doc]

| primers |  |  |
| --- | --- | --- |
|  |  |  |
| Tyr1680Phe *forward* | 5’ GATTTTGACATTTTTGATGAGGATG 3’ | |
| Tyr1680Phe *reverse* | 5’ CATCCTCATCAAAAATGTCAAAATC 3’ | |
| Ser2119Tyr *forward* | 5’ CAGACTTATCGAGGAAATTACACTGGAACCTTAATG 3’ | |
| Ser2119Tyr *reverse* | 5’ CATTAAGGTTCCAGTGTAATTTCCTCGATAAGTCTG 3’ | |
| Arg2150His *forward* | 5’ ATTATTGCTCGATACATCCATTTGCACCCAACTCAT 3’ | |
| Arg2150His *reverse* | 5’ ATGAGTTGGGTGCAAATGGATGTATCGAGCAATAAT 3’ | |
| Del2201 *forward* | 5’ ACCAATATGTTTACCTGGTCTCCTTCAAAAGCTCGA 3’ | |
| Del2201 *reverse* | 5’ TGAAGGAGACCAGGTAAACATATTGGTAAAGTAGGA3’ | |
| Pro2300Ser *forward* | 5’ GTGAACTCTCTAGACCCATCGTTACTGACTCGCTAC 3’ | |
| Pro2300Ser *reverse* | 5’ GTAGCGAGTCAGTAACGATGGGTCTAGAGAGTTCAC 3’ | |
| Deletion YFP *forward* | 5’ AGAAGCTTCTCCCAGAATCCACCAGTCTTGAAACGC 3’ | |
| Deletion YFP *reverse* | 5’ GCGTTTCAAGACTGGTGGATTCTGGGAGAAGCTTCT 3’ | |
